# Personal

**Published:** 1902-08

**Authors:** 


### PERSONAL.

Dr. George H. Simmons, of Chicago, secretary of the American Medical Association and editor of its journal, recently submitted to an operation for gallstone disease which resulted favorably, a large calculus obstructing the cystic duct having been removed. This apparently accounts for Dr. Simmons's ill health and from which he is now expected to recover. The Journal extends its sympathy and best wishes for his prompt restoration to health.

Colonel William H. Forwood, M. D., assistant surgeon-general, was promoted to be Surgeon-General U. S. Army, vice Brig.-General George M. Sternberg, M.D., retired June 7, 1902. General Forwood has distinguished himself in two wars and will retire September 7, 1902, after but three months' service as the head of the medical department of the army.

Colonel Robert Maitland O'Reilly, M. D., chief medical officer, Department of California, has been designated by the President to act as surgeon-general on and after the retirement of General Forwood. Col. O'Reilly was a medical cadet during the civil war and was appointed assistant surgeon in the army in April, 1867. He has seen active service on the plains and was a chief medical officer with the rank of lieutentant-colonel during the war with Spain.

Dr. R. T. Rolph, who has been in medical charge of a sanitarium in Arizona for two or three years, has returned to Dunkirk and resumed the practice of his profession. He will reside, as before, at Fredonia with offices at Dunkirk. Dr. Rolph looks in robust health, and remarked during a recent visit to Buffalo that he had been greatly benefited by his residence in the genial climate of Arizona.

Dr. John Halliday Croom, of Edinburgh, has received the honor of knighthood at the hands of King Edward VII. in consideration of his distinguished career in medicine. Sir John is an honorary fellow of the American Association of Obstetricians and Gynecologists and the association tenders him its hearty congratulations as a deserving recipient of coronation honors.

Dr. William A. Moore, of Binghamton, vice-president of the Medical Society of the State of New York, recently paid a visit to Buffalo in the interests of the society, and was the guest of Dr. Henry Reed Hopkins. Dr. Moore is taking an active part in promoting the welfare of the society of which he is the second officer, and which is rapidly approaching its centennial anniversary.

---

Dr. Julius Pohlman, of Buffalo, sailed July 12, to spend his vacation in Europe. Other Buffalo physicians abroad are Drs. Max Breuer, Sydney A. Dunham, Herman Mynter, Alexander M. Blair, Allen A. Jones, and Roswell Park. Dr. and Mrs. Mann will sail August 6.

---

Dr. M. J. Lewi, of New York, secretary of the State Board of Medical Examiners, attended the annual convocation of the University of the State of New York, at Albany, July 1 and 2, 1902, and participated in the discussions.

---

Dr. Roswell Park, of Buffalo, sailed early in July for a five weeks' tour in Europe. Dr. Park was the recipient of the honorary degree of Doctor of Laws from Yale University at its last annual commencement.

---

James Russell Parsons, Jr. Esq., secretary of the University of the State of New York, received the honorary degree of Doctor of Laws at the annual commencement of Trinity College in June.

---

Dr. Daniel Lewis, of New York, was elected president of the American Congress of Tuberculosis at its annual meeting held at New York, June 3-5, 1902.

---

Dr. John H. Grant, of Buffalo, was elected medical director of the department of New York, G.A.R. during the recent encampment at Saratoga.

